# Successful Video‐Assisted Thoracoscopic Management of Rare Thoracic Complications After Percutaneous Biliary Drainage: A Report of Two Cases

**DOI:** 10.1111/ases.70105

**Published:** 2025-06-24

**Authors:** Kazuki Hayashi, Yusuke Kita, Jun Hanaoka

**Affiliations:** ^1^ Department of General Thoracic Surgery, Omi Medical Center Kusatsu Shiga Japan; ^2^ Division of General Thoracic Surgery, Department of Surgery Shiga University of Medical Science Otsu Shiga Japan

**Keywords:** drainage, gallbladder, video‐assisted thoracoscopic surgery

## Abstract

Percutaneous transhepatic gallbladder drainage and percutaneous transhepatic cholangiodrainage are effective therapeutic options for biliary disease, although they occasionally result in thoracic complications. We report two cases of thoracic complications that developed after biliary drainage and were successfully treated using video‐assisted thoracoscopic surgery. The first case involved a 55‐year‐old man who developed a chest wall (extrapleural) abscess after percutaneous transhepatic gallbladder drainage and laparoscopic cholecystectomy. Video‐assisted thoracoscopic surgery revealed an extrapleural abscess that was initially diagnosed as empyema. The second case involved a 71‐year‐old woman who developed bilious pleural effusion after percutaneous transhepatic cholangiodrainage. Video‐assisted thoracoscopic surgery was used to identify and close a diaphragmatic fistula. Both patients had a favorable postoperative course. This report demonstrates that video‐assisted thoracoscopic surgery can be an effective therapeutic approach for thoracic complications after percutaneous biliary drainage.

## 1 Introduction

Percutaneous transhepatic gallbladder drainage (PTGBD) and percutaneous transhepatic cholangiodrainage (PTCD) are effective treatment methods for conditions such as cholecystitis, obstructive jaundice, and suppurative cholangitis [1, 2]. However, these procedures can occasionally lead to thoracic complications [3, 4, 5, 6, 7, 8, 9]. We report two cases in which a chest wall (extrapleural) abscess and biliary pleuritis developed after PTGBD and PTCD, respectively. Both cases were successfully managed using video‐assisted thoracoscopic surgery (VATS).

## 2 Case Presentation

### 2.1 Case 1

**FIGURE 1:**
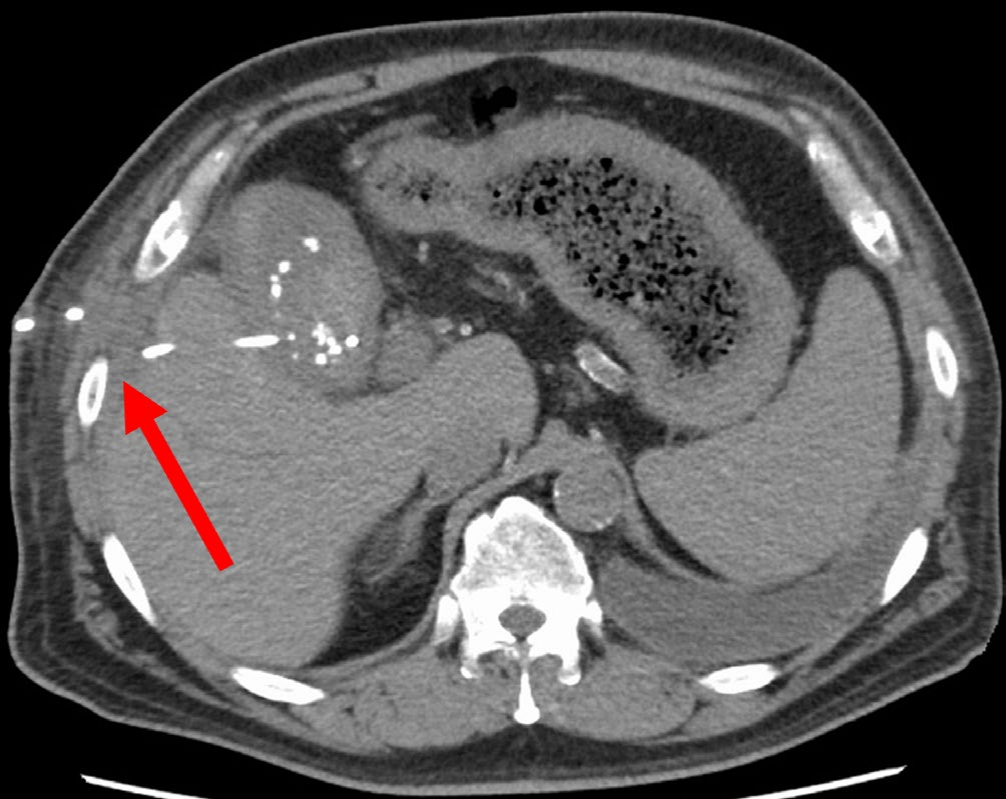
Case 1. Abdominal computed tomography image taken after percutaneous transhepatic gallbladder drainage placement at the previous hospital. The catheter is placed through the eighth intercostal space (arrow).

A 55‐year‐old man with a history of diabetic nephropathy, who was on maintenance hemodialysis and had ischemic heart disease status following coronary artery stenting and bypass grafting, underwent ultrasound‐guided PTGBD through the eighth intercostal space for acute cholecystitis at another hospital (Figure 1). Despite continued antibiotic therapy, the cholecystitis did not improve, and the patient underwent laparoscopic cholecystectomy 36 days after PTGBD placement.

**FIGURE 2:**
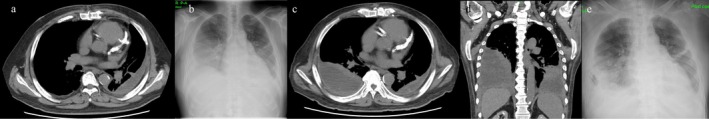
Case 1. (a) Chest computed tomography (CT) after percutaneous transhepatic gallbladder drainage placement at the previous hospital. No right pleural effusion was observed at this time. (b) Chest radiograph at the time of referral to our hospital. Apparent right pleural effusion is visualized. (c) Chest CT at the time of referral to our hospital. Fluid collection predominantly in the right hemithorax appears to be pleural effusion. Left pleural effusion remains unchanged compared with the previous CT at the referring hospital. (d) Coronal view of image (c). (e) Immediate postoperative photograph. Drainage tubes were placed in both the abscess cavity and pleural space.

Postoperatively, the patient developed right‐sided pleural effusion that did not respond to conservative management. He was referred to our hospital with suspected empyema for surgical intervention (Figure 2a–d). Thoracic drainage was not performed at the referring hospital.

During VATS, a small amount of serous pleural fluid was observed. When approaching through a separate incision, a large amount of brownish fluid was evacuated. Further exploration revealed that the fluid was not collecting within the pleural cavity but in the extrapleural space, indicating a chest wall abscess rather than empyema. The parietal pleura was incised to create communication between the abscess cavity and pleural space, followed by thorough debridement and irrigation (Video S1) (Figure 2e). Cultures of the abscess fluid were negative. Postoperatively, fluid re‐accumulation was not observed in the chest wall, and the patient was transferred to the referring hospital for rehabilitation on postoperative Day 21.

### 2.2 Case 2

**FIGURE 3:**
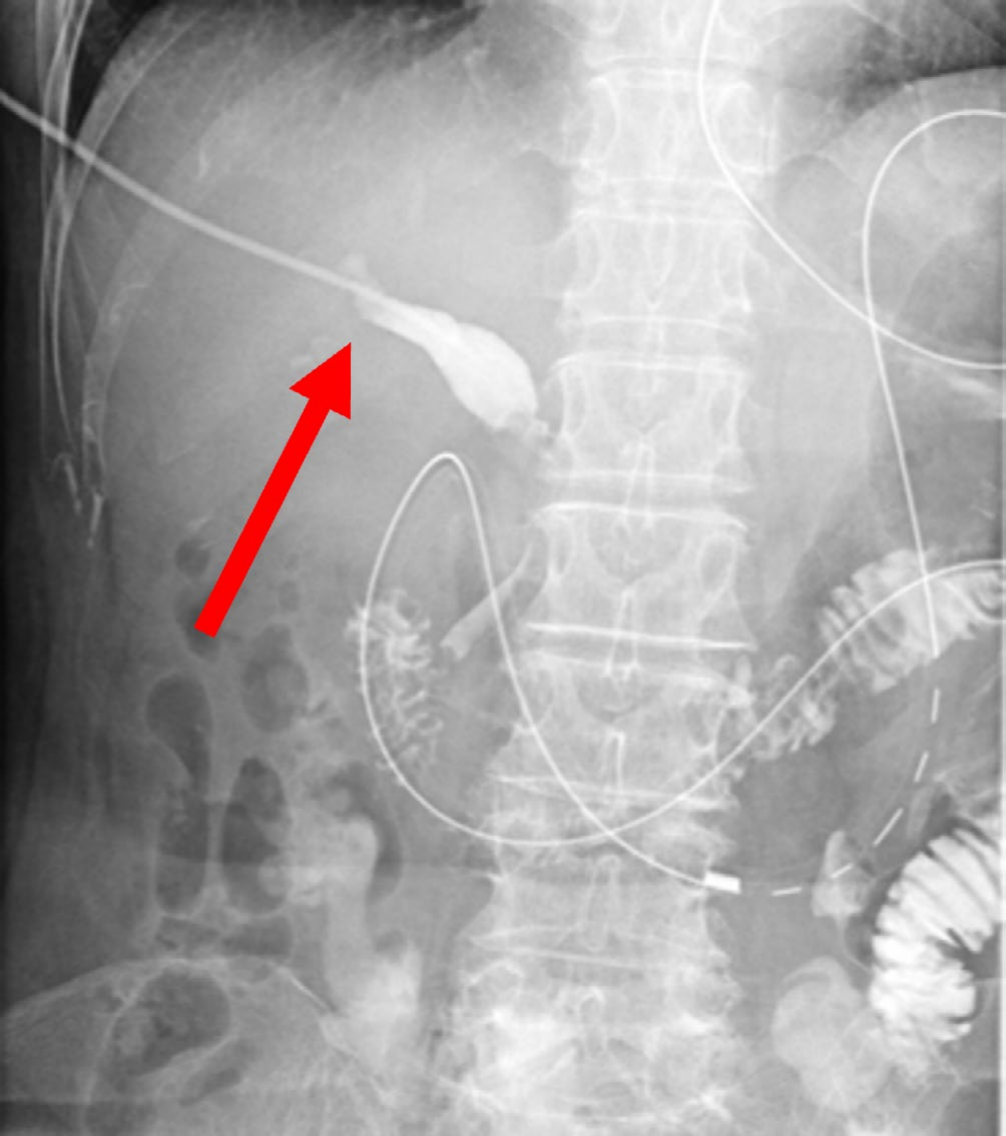
Case 2. Fluoroscopic image taken during percutaneous transhepatic cholangiodrainage placement. The catheter has advanced into the bile duct and contrast enhancement is visible (arrow).

A 71‐year‐old woman with gallbladder cancer that had invaded the duodenum and caused intestinal obstruction was scheduled for palliative bypass surgery. Owing to the development of jaundice caused by common bile duct stenosis, the patient underwent ultrasound‐guided PTCD (Figure 3). Four days after PTCD, during planned biliary stent placement, surgeons discovered that the catheter tip had accidentally migrated into the peritoneal cavity. The patient refused a repeat biliary drainage procedure and underwent gastrointestinal bypass surgery 8 days after the initial PTCD.

**FIGURE 4:**
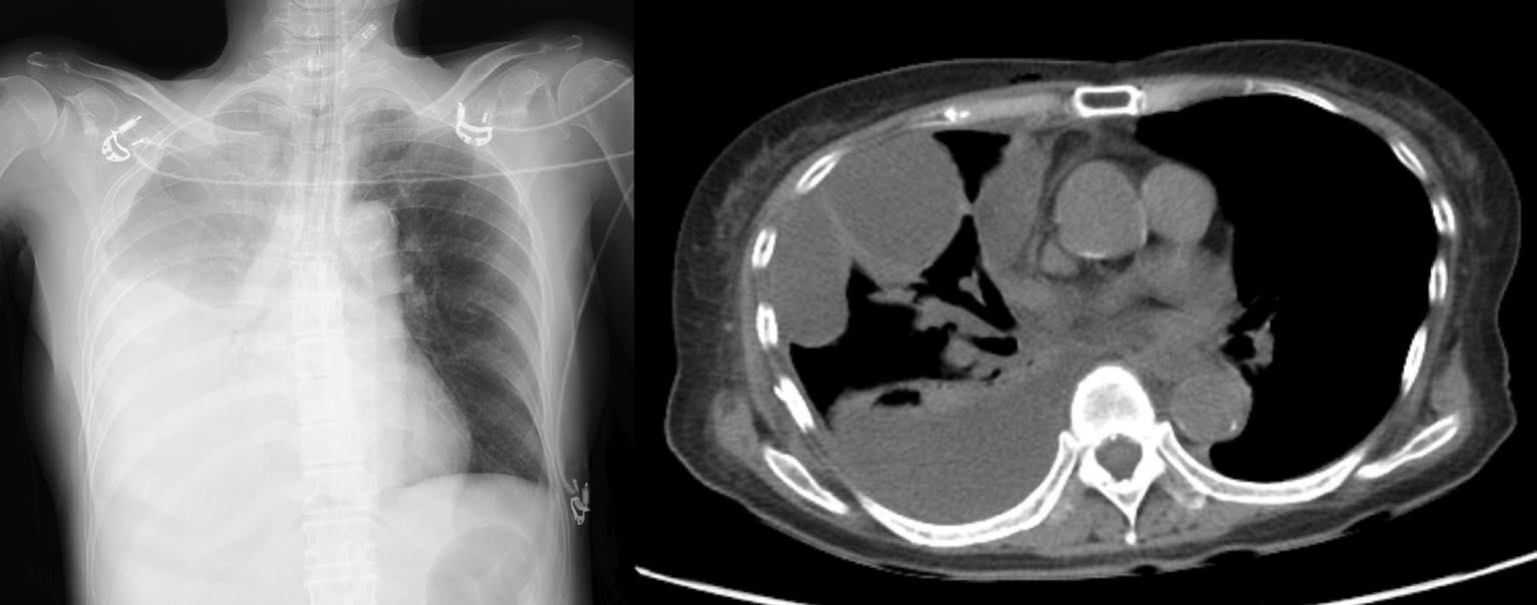
Case 2. Postoperative chest radiograph after palliative gastrointestinal bypass surgery showing a marked increase in right pleural effusion. During subsequent follow‐up, the right pleural effusion did not improve, and computed tomography confirmed multiloculated effusion.

Postoperative imaging revealed a large, right‐sided pleural effusion (Figure 4). Thoracic drainage yielded bilious pleural fluid. Despite antibiotic therapy, bilious drainage persisted, fluid collection became multiloculated, and the patient experienced severe right‐sided chest pain. Bile migration into the thoracic cavity via the diaphragm was suspected, and the patient was referred to our department.

VATS revealed that the thoracic cavity was filled with bilious pleural fluid and fibrinopurulent membranes. After extensive debridement and decortication, examination of the diaphragmatic surface revealed a single fistulous tract that was closed using sutures (Video S2) (the other visible suture was made to repair a tear in the diaphragmatic surface that occurred during port placement). An additional drain was placed in the peritoneal cavity through the right subcostal region. Postoperatively, bilious pleural drainage ceased, and the patient's pain improved remarkably. The patient underwent subsequent biliary stent placement and was discharged 17 days after VATS.

## 3 Discussion

Case 1 initially presented with suspected empyema following cholecystitis that was found to be a chest wall (extrapleural) abscess. Case 2 involved diaphragmatic injury during PTCD for obstructive jaundice due to gallbladder cancer, resulting in bile leakage from the displaced biliary catheter into the peritoneal cavity and subsequent migration into the thoracic cavity, causing biliary pleuritis. Although PTGBD and PTCD are common therapeutic procedures for biliary diseases, the thoracic complications associated with these procedures are relatively rare. The incidence of peritonitis due to catheter displacement after PTGBD is 2.5% [9]. In contrast, although empyema and biliopleural fistulas have occasionally been reported [3, 4, 5], their incidence is presumed to be lower than that of peritonitis. However, once these complications develop, they carry the risk of a prolonged clinical course and increased severity, making accurate diagnosis and appropriate therapeutic intervention essential.

A notable aspect of Case 1 was that the lesion, initially interpreted as a pleural effusion on preoperative imaging, was actually an extrapleural abscess (lateral to the parietal pleura) (Additional Figure). Although empyema has been reported after biliary drainage [3, 4, 5], to the best of our knowledge, chest wall (extrapleural) abscesses have not previously been reported in this context. Dropped or retained gallstones are potential causes of postcholecystectomy empyema and chronic cutaneous chest wall fistula [6, 7, 8]; however, no evident gallstones were identified on imaging in our patient. Although attribution of the abscess to either PTGBD or cholecystectomy is difficult, a retrospective review revealed continuity between the abscess cavity and the eighth intercostal space where the PTGBD was performed; this suggests that this complication was likely related to the PTGBD procedure. Furthermore, the patient's underlying conditions, including diabetes and maintenance hemodialysis, likely contributed to his susceptibility to infections and abscess formation.

In Case 2, diaphragmatic injury during PTCD led to biliary pleuritis. Although local anesthesia‐based closure of similar fistulae has been reported [10], we opted for VATS under general anesthesia considering the patient's preferences and need for definitive fistula closure. This approach enabled thorough thoracic cavity exploration and irrigation in addition to secure fistula closure.

VATS was an effective treatment method in both cases. It offers minimally invasive access, provides excellent visualization, and enables appropriate intervention. VATS represents a valuable therapeutic option for thoracic complications that develop after biliary drainage procedures and fail to respond to conservative management using antibiotic therapy.

In conclusion, although uncommon, thoracic complications after percutaneous biliary drainage procedures require prompt recognition and appropriate management. VATS offers a minimally invasive yet effective approach for both diagnostic clarification and therapeutic intervention in challenging cases. Surgeons should be aware of the potential thoracic complications following PTGBD and PTCD, particularly in patients with predisposing risk factors.

## Author Contributions

K.H., Y.K., and J.H. performed the surgery and provided postoperative care to the patients. K.H. was a major contributor in the writing of the manuscript. K.H., Y.K., and J.H. contributed to data collection and interpretation and critically reviewed the manuscript. All the authors have read and approved the final version of the manuscript.

## Ethics Statement

The study was conducted in accordance with the ethical principles outlined in the Declaration of Helsinki. Patient anonymity was maintained, and personal information was kept confidential.

## Consent

Informed consent was obtained from the patients.

## Conflicts of Interest

The authors declare no conflicts of interest.

## Supporting information


**Figure S1.** Schematic diagram corresponding to coronal CT image of this case. Red line represents parietal pleura, illustrating that the abscess was located external to the parietal pleura.

**Video S1.** Case 1. The thoracoscope was moved from the incision while observing the thoracic cavity to another incision to examine the interior of the abscess cavity. When pressure was applied from the abscess side toward the thoracic cavity and observed from within the thoracic cavity, movement of the parietal pleura was visible. This confirmed that the abscess was located external to the pleural space. The parietal pleura was incised to create communication between the abscess cavity and the pleural space, allowing for continued drainage and irrigation.

**Video S2.** Case 2. Thoracic cavity contains bilious pleural fluid and fibrinopurulent membranes. After thorough debridement, examination of the diaphragmatic surface reveals a fistulous tract. This site is ligated and closed (the other visible suture repaired a tear in the diaphragmatic surface that occurred during port placement).

## Data Availability

Data sharing is not applicable to this report because no new data were created or analyzed in this report.
