# Supplementary figures and images for: Successful Video‐Assisted Thoracoscopic Management of Rare Thoracic Complications After Percutaneous Biliary Drainage: A Report of Two Cases
Source: Asian J Endosc Surg. 2025 Jun 24;18(1):e70105. doi: 10.1111/ases.70105 (PMC12188032; doi:10.1111/ases.70105)

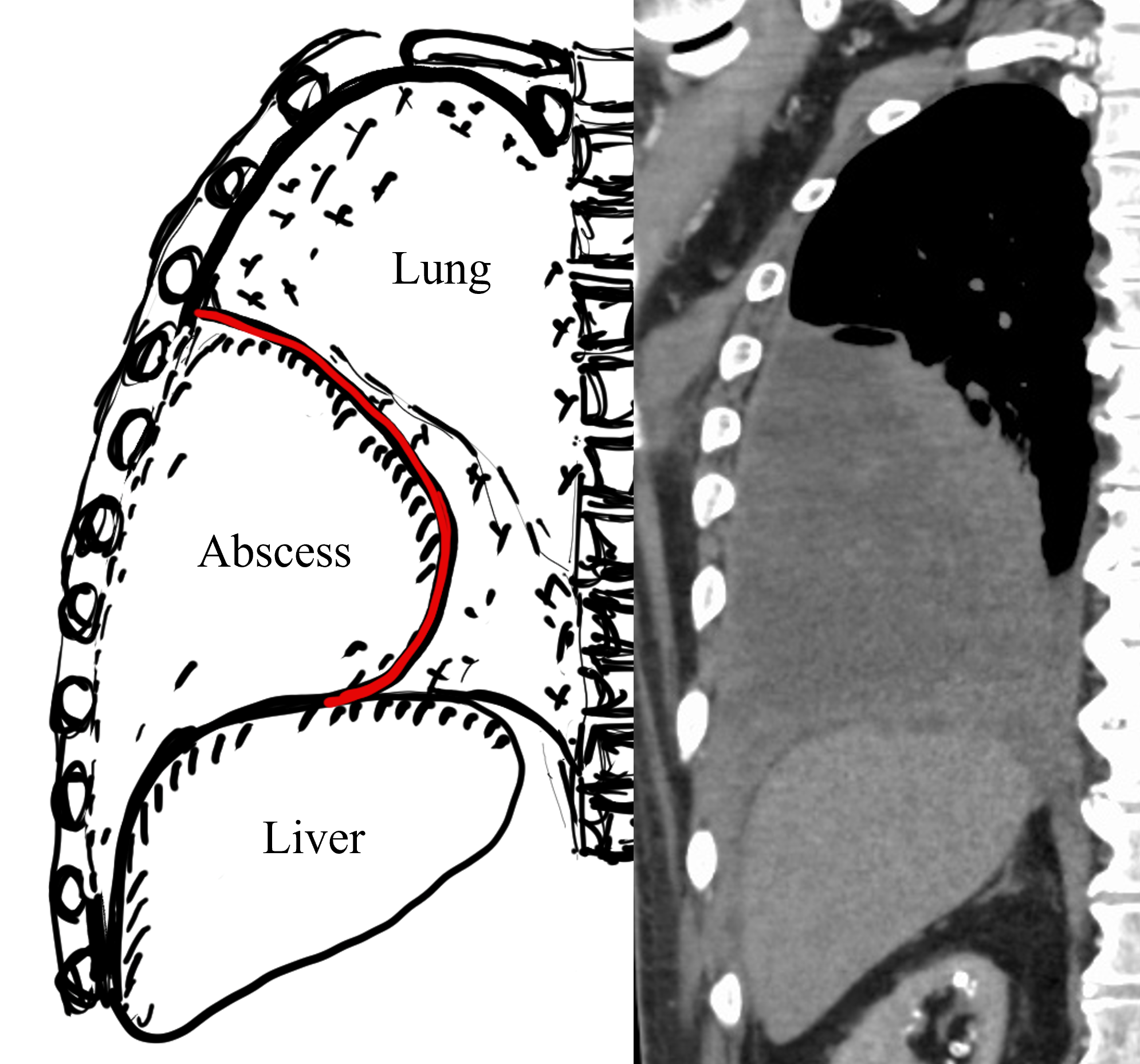

Supplement: Supplementary file 1 — Figure S1. Schematic diagram corresponding to coronal CT image of this case. Red line represents parietal pleura, illustrating that the abscess was located external to the parietal pleura. [file ASES-18-e70105-s002.tif]
